# Antibiotic Treatment Protocols and Germ-Free Mouse Models in Vascular Research

**DOI:** 10.3389/fimmu.2019.02174

**Published:** 2019-09-12

**Authors:** Franziska Bayer, Stefanie Ascher, Giulia Pontarollo, Christoph Reinhardt

**Affiliations:** ^1^Center for Thrombosis and Hemostasis (CTH), University Medical Center Mainz, Johannes Gutenberg University Mainz, Mainz, Germany; ^2^German Center for Cardiovascular Research (DZHK), Partner Site RheinMain, Mainz, Germany

**Keywords:** platelets, germ-free mouse models, antibiotics, thrombosis, microbiota, vascular function

## Abstract

The gut microbiota influence host vascular physiology locally in the intestine, but also evoke remote effects that impact distant organ functions. Amongst others, the microbiota affect intestinal vascular remodeling, lymphatic development, cardiac output and vascular function, myelopoiesis, prothrombotic platelet function, and immunovigilance of the host. Experimentally, host-microbiota interactions are investigated by working with animals devoid of symbiotic bacteria, i.e., by the decimation of gut commensals by antibiotic administration, or by taking advantage of germ-free mouse isolator technology. Remarkably, some of the vascular effects that were unraveled following antibiotic treatment were not observed in the germ-free animal models and *vice versa*. In this review, we will dissect the manifold influences that antibiotics have on the cardiovascular system and their effects on thromboinflammation.

## Introduction

During the past decade, microbiome research has started to explore how the densely colonized gut resident ecosystem (microbiota) affects the host's vascular physiology (1, 2). This symbiotic microbial community, whose composition is highly dependent on nutrition, interferes with host metabolism and constitutes a chronic inflammatory stimulus. Nowadays, the various influences of the gut microbiome on vascular inflammatory phenotypes, such as atherosclerosis, myocardial infarction, arterial thrombosis, and stroke, are being increasingly recognized (3–6). Moreover, recent research with germ-free (GF) mouse isolator technology revealed a reduced tendency of arterial thrombus formation in different carotid artery mouse thrombosis models, arguing for a contribution of the gut microbiota to thromboinflammation (7–9).

Indeed, mounting evidence is linking the gut microbiota to the onset of cardiovascular disease and arterial thrombosis (10–12). Among microbial-associated molecular patterns (MAMPs), the microbiota-derived choline-metabolite trimethylamine (TMA) that is produced by gut bacterial TMA-lyases (cutC) and targets the liver, is just emerging as a risk factor for thrombotic manifestations (8, 13–15). Through the action of flavin-containing monoxygenase-3 (FMO3), TMA is oxidized into trimethylamine N-oxide (TMAO) (16), a metabolite that was reported to relieve agonist-induced platelet activation (7). Remarkably, both elevated TMAO plasma levels and the activation of Toll-like receptor (TLR) signaling by MAMPs were shown to accelerate atherogenesis in the apolipoprotein E (Apoe)-deficient mice, which are currently used as an animal model of atherosclerosis (17, 18). Clearly, the influences of the microbiota on atherosclerosis are not limited to the TMAO pathway and pattern-recognition receptors, as additional microbiota-derived factors, such as short chain fatty acids (SCFA), were recently identified (19).

Intriguingly, current research, investigating the impact of the gut microbiota on vascular phenotypes, has revealed discrepancies between broad-spectrum antibiotic treatment protocols and investigations on GF mouse models. To give an example, antibiotic treatment of (*Apoe*)-deficient mice starting at weaning (4 weeks of age) resulted in a reduced development of aortic root lesions at 20 weeks of age, when the mice were fed with a 1.0% choline-rich chow diet (17). Likewise, Kasahara et al. reported reduced aortic root plaque areas together with reduced macrophage and CD4^+^ cell infiltration of the aortic sinus, when studying GF *Apoe*-deficient mice kept on an irradiated chow diet and analyzed at 20 weeks of age (20). To complicate the picture, the aortic root lesion size of GF *Apoe*-deficient mice kept for 12 weeks on 1.2% choline-rich chow diet starting at the age of 8 weeks was not different compared to conventionally raised (CONV-R) control mice (21). In the study conducted by Wright et al. no differences in aortic root lesion size was reported when GF and CONV-R *Apoe*-deficient mice were fed with a Western diet at weaning until 22 weeks of age (22). Finally, similar to the study of Lindskog Jonsson et al., Stepankova et al. reported increased atherosclerotic lesion sizes in the thoracic aortas of GF *Apoe*-deficient mice after feeding with a low cholesterol diet for 3–4 months (3, 21). These seemingly controversial results could be explained by apparently minor differences in the experimental protocols, e.g., the mouse line studied, the various diets used, the feeding time scheme applied, or the normalization of the measured lesion sizes. On the other hand, it is becoming increasingly clear that the interpretation of data collected after microbiota decimation experiments by antibiotics should be considered with caution, given the experimental variables and the several side effects of antibiotic treatment protocols. It should also be kept in mind that antibiotic decimation of the microbiota using broad-spectrum antibiotics represents a selective pressure that favors overgrowth of resistant bacterial taxa (23), which could in principle be causative at least for some of the described outcomes.

Moreover, recent research has implicated the intestinal microbiota in arterial thrombosis and it has been proposed that selective inhibition of the TMA-generating gut microbial enzymes could lower thrombotic risk (24). While TMAO has been shown to facilitate agonist-induced platelet activation (7), it was demonstrated that microbiota-derived TLR-ligands promote the activation of hepatic endothelial cells, triggering von Willebrand factor (VWF) synthesis and release (8, 15, 25). In mouse models, both pathways promote carotid artery thrombosis and thrombus growth was reduced in the GF mouse model, linking the gut microbiota mechanistically to arterial thrombosis (7, 8, 26). In the literature, in addition to atherosclerotic phenotypes, there are numerous other examples on microbiota-dependent vascular phenotypes that differ between gnotobiotic mouse models and depletion of the gut microbiota with antibiotics.

Here, we provide an overview on GF mouse isolator technology and antibiotic treatment protocols for microbiota depletion that are widely used to study vascular phenotypes. We will explain the limitations of these mouse models, but also describe the recently gained insights on microbiota-driven influences, affecting vascular physiology, cardiovascular disease development and thromboinflammation.

## Differences and Similarities of Germ-Free and Antibiotic-Treated Mice

In 1885, Louis Pasteur claimed that a life without microbial associates is not possible (27). Several years later, in the 1940s, the first colonies of GF rodents were established. Since its beginning, the usage of this technology became a valuable model to understand how the microbiota impacts host physiology and disease processes (28). Gnotobiotic animals such as mice colonized with Altered Schaedler Flora (a defined bacterial community) or germ-free (axenic) mice, lacking all microorganisms, are animal models characterized by a defined colonization status (29). In particular, GF mice are bred and kept for their whole lifetime in sterile isolators to prevent their exposure to microorganisms. These animals are a biological model system to either study the complete absence of microbes, or to investigate the effects of colonization with selected and known microbial species (e.g., in the mono-association experiment) (30). However, the GF mouse model is a labor-intensive technology, which requires permanent controls for the hygiene status of the isolators and special facilities (30, 31). As an alternative and more basic method for the depletion of microbiota, administration of broad-spectrum antibiotics is commonly used. In contrast to GF mice, antibiotic application does not lead to the depletion of all microbes, but can selectively deplete different members of the gut microbiota (32). Furthermore, to prevent dehydration during treatment, Reikvam et al. recommended to gavage mice instead of delivering the antibiotics in the drinking water, or to combine both applications (33).

**Figure 1:**
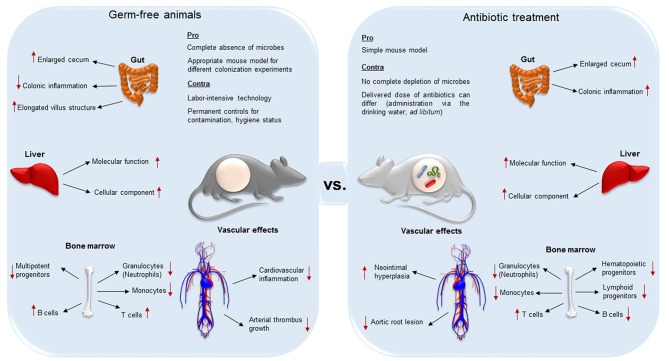
Differences and similarities of germ-free (axenic) and broad-spectrum antibiotic-treated mice (decimation of microbes). While both techniques result in the aberrant enlargement of the cecum, GF mice are less susceptible for colonic inflammation, and present elongated villus structures compared to antibiotic treated animals. In both the animal models, absence of gut microbiota alters protein expression levels in the liver. In the bone marrow, the two mouse models present both reduced granulocytes, monocytes and progenitor cells, but higher T cell levels. On the other hand, while B cells in GF animals are increased, in antibiotic-treated counterparts they were reported to be decreased. Both the complete absence and the decimation of the gut microbiota influences vascular physiology and have an effect on vascular disease. Neointimal hyperplasia, a proliferation and inflammation response to arterial injury, was increased in antibiotic treated rats. Antibiotic treatment leads to a diminished development of aortic root lesion. Additionally, germ-free mice were protected from cardiac inflammation and arterial thrombus growth.

Importantly, decimation of the microbiota affects the anatomy and function of various organs such as liver and gut (34, 35) (summarized in Figure 1). One of the most evident anatomical changes is the enlarged cecum, observed both in GF and antibiotic-treated mice (30, 33). Furthermore, GF mice present elongated villus structures, a reduced villus width, and poorly developed capillary networks in small intestinal villi (1, 36, 37). In addition to altered organ morphology and physiology, immune cell populations are influenced by antibiotic treatment (38–40). Because the GF animal model and antibiotic decimation of the microbiota may result in different vascular phenotypes with respect to anatomy and function (8, 17, 41, 42), it is important to be aware of the limitations of these experimental models that enable the exploration of microbiota-host interactions.

## Antibiotics and Their Influence on Myeloid Cell Function

In addition to the resulting selection of resistant bacterial taxa (23), antibiotics severely influence the development of the myeloid cell lineage (40), which may greatly hamper the interpretation of results on vascular physiology. It is firmly established that long-term treatment with beta-lactam antibiotics results in an inhibition of granulopoiesis (43). In this clinical study, the presence of granulocyte precursors with the lack of well-differentiated myeloid cells was observed in human bone marrow aspirates, discussed by the authors as a myelotoxic effect of beta-lactam antibiotics. Most important, in bone marrow cultures, Neftel et al. described a direct inhibitory effect of beta-lactam antibiotics on *in vitro* granulopoiesis. In addition, thrombocytopenia caused by beta-lactam antibiotic treatment was remarked. Likewise, reduced neutrophil counts were reported in the bone marrow of long-term antibiotics treated mice, which was explained by neutrophil aging promoted by MAMPs derived from the gut microbiota (40).

In contrast, Hergott et al. demonstrated that antibiotic treatment accelerates the turnover of circulating neutrophils and inflammatory monocytes, which showed decreased cell counts in the blood, the bone marrow and the spleen (44). In this work, it was described that long-term antibiotic treatment (i.e. 3–4 weeks) with the frequently used cocktail of neomycin, vancomycin, metronidazole, and ampicillin for microbiota decimation was associated with reduced survival of neutrophils and inflammatory monocytes, which was confirmed in the GF mouse model. This phenotype was explained by microbiota-induced sensing of peptidoglycan of nucleotide-binding oligomerization domain-containing protein-1 (NOD1), and by the production of interleukin-17A (IL-17A) in the ileum (44). The requirement of NOD1 sensing in mesenchymal stromal cells in the reduction of immune cells survival was confirmed in a subsequent study with GF mice, showing that these models presented an overall reduced numbers of hematopoietic stem and multipotent progenitor cells (39). By using the same antibiotic cocktail over 14 days and an alternative antibiotic cocktail containing ciprofloxacin and metronidazole, Josefsdottir et al. demonstrated that not only myeloid cell numbers were dramatically reduced, but also the peripheral blood counts of various lymphocyte subsets along with the counts of hematopoietic progenitors were diminished (B-cells, CD4^+^ T-cells, CD8^+^ T-cells) (38). Interestingly, it was demonstrated that the cell cycle activity in hematopoietic stem cells and myeloid progenitors was increased in the antibiotic treated group, excluding a direct myelotoxic effect of the antibiotics used by murine bone marrow culture experiments. Of note, there was no antibiotic effect on the hematopoietic progenitor population and on granulocytes in signal transducer and activator of transcription 1 (Stat1)-deficient mice, indicating that upstream pathways of this transcription factor may play a critical role.

While the obvious question for the fundamental differences between antibiotic treatment and GF housing state with regard to hematopoietic functions is apparent, these important studies clearly demonstrate that antibiotic intervention has manifold effects on innate immune cell types, which may lead to inconclusive mechanistic data in hematologic research. Therefore, gnotobiotic models based on GF knock-out mouse strains are essential to control for the validity of results obtained by antibiotic treatment protocols.

## Antibiotics and Their Influence on Clotting, Platelet Function and the Vasculature

### Antibiotic Treatment Interferes With Vitamin K Metabolism and Clotting Factors

The lipophilic essential vitamin K has two sources: it can be found in green leafed plants as phylloquinone (vitamin K1) and as the microbial metabolite menaquinone (vitamin K2), which is produced by several bacterial species (45–48). Synthesis of clotting factors II, VII, IX, and X and their post-translational modifications in the liver are vitamin K-dependent (45, 46). The correlation between antibiotic treatment and increased bleeding risk due to vitamin K deficiency was already described in 1952 by Dam et al. (47). Over the years, more case reports of hypoprothrombinemic bleeding were published, involving several classes of antibiotics (46, 48–50).

In this context, since 1980s, N-methylthiotetrazole (NMTT) cephalosporins (for example cefoperazone, cefotetan, moxalactam, and cefamandole) administration has been frequently reported to result in impaired hemostasis (45, 46, 50–52). Here, several mechanisms and speculations are discussed in the literature, including: (a), interference of NMTT-side chain with vitamin K metabolism (45), (b), indirect inhibition of vitamin K-dependent blood coagulation (50, 53), and (c), reduction of menaquinone producing bacteria (50, 53). A more recent publication by Fotouhie et al. supports in part hypothesis (c), stating that several risk factors have to concur for the appearance of clinical symptoms (52). The following risk factors were identified: insufficient dietary intake of phylloquinone, modification of normal gut microbial communities via antibiotics, malabsorption of vitamin K, or chronic liver disease (48). Hence, antibiotic treatment protocols may interfere with the synthesis of vitamin K-dependent blood clotting factors.

### Antibiotic Treatment and Platelets

Other reasons for impaired hemostasis and thrombus formation following antibiotics intake are thrombocytopenia or impaired platelet function. In the early 1970s, the antibiotic ristocetin got withdrawn from clinical use because it induced platelet aggregation (54). As a result, many research groups then replaced ristocetin administration with vancomycin, as the two molecules share many chemical properties. However, even if vancomycin itself is not able to directly trigger platelet aggregation, it was reported to induce precipitates in which platelets are incorporated (55, 56). A more recent publication revealed the underlying mechanism for vancomycin-induced thrombocytopenia. Towhid et al. described platelet apoptosis paralleled by mitochondria depolarization, activation of caspase-3, cell membrane scrambling and ceramide formation. Further tests revealed that Ca^2+^ is necessary for vancomycin to cause these effects. They hypothesized that the cell scrambling induces an accelerated clearance of platelets from the blood, resulting in the observed thrombocytopenia. Importantly, all the described side effects are induced by exploiting vancomycin concentrations normally reached during standard protocols of antibiotic treatments (57).

Penicillin antibiotics may also have an effect on platelets. Clinically relevant concentrations of penicillin G and carbenicillin seem to have a global effect on platelet membrane receptors, as platelets become less responsive to physiologic agonists and fail to aggregate with bovine factor VIIIa stimulation (45). Subsequently, Pastakia et al. have shown that penicillin G inhibits thrombin-induced upregulation of GPIb-IX levels on the platelet surface (58, 59). In contrast, other beta-lactam antibiotics (ceftriaxone, ceftazidime, and aztreonam) did not inhibit platelet aggregation (60). On the contrary, ceftriaxone (and to a lesser extent aztreonam) was shown to enhance platelet aggregation, but the underlying molecular mechanisms remain unclear. Another antibiotic presenting inhibitory effect on *in vitro* platelet aggregation, even after oral treatment, is metronidazole (61).

The underlying mechanisms for thrombocytopenia (i.e., a circulating platelet count inferior to 150,000/mm^3^) are increased platelet consumption/destruction or reduced platelet production. Linezolid, an antibiotic commonly used to treat vancomycin-resistant enterococci and methicillin-resistant *Staphylococcus aureus* infections, is known to induce myelosuppression, but is more frequently associated with thrombo- than pancytopenia (62). It was described that linezolid has no direct toxic effects on platelets and it does not affect the differentiation of hematopoietic stem cells, but via phosphorylation of an enzyme relevant for platelet release, it induces thrombocytopenia (62). Although some influences of antibiotics on platelet functions were identified, the information on effects of antibiotic treatment on the coagulation system remains sparse.

### Antibiotic Treatment and Vasomodulatory Effects

Since the 1950s, aminoglycoside antibiotics are known to have hypotensive effects and a negative inotropic effect on the heart (63). Gentamicin was reported to cause hypotension resulting from vasodilatation and relaxation of smooth muscle cells, thus yielding reduced vascular resistance, together with decreased cardiac contraction force and bradycardia. The proposed mechanism is an impaired Ca^2+^ influx. Neomycin, gentamicin and, to a lesser extent, streptomycin, and kanamycin were demonstrated to have vasorelaxant effects on the cerebral arteries of dogs (64). All four antibiotics inhibited vasoconstriction after administering depolarizing concentrations of potassium chloride (64). Two mechanisms were proposed: (a), the inhibition of phospholipase C (an enzyme that catalyzes the production of the second messenger molecule inositol trisphosphate, which drives the release of Ca^2+^ from the sarcoplasmatic reticulum) and (b), direct interference with Ca^2+^ influx by blocking L-type voltage-dependent Ca^2+^ channels (64). Belus et al. revealed the mechanism for the aminoglycoside antibiotics neomycin, gentamicin, and streptomycin negative inotropic effect. The resting and transient intracellular Ca^2+^ levels of rat ventricular myocytes are decreased, leading to reduced contractility (65). Furthermore, high dosages of vancomycin (glycopeptide antibiotic) and tobramycin (aminoglycoside antibiotic) have relaxing effects on the vascular smooth muscles (66). Not only aminoglycoside antibiotics, but also beta-lactam antibiotics seem to have an influence on Ca^2+^-influx in the vascular endothelium (60). This indicates that vascular function is significantly affected by the administration of several different antibiotics.

## Conclusions

GF mouse models and antibiotic treatment protocols are frequently used as comparable methods to investigate the effect of gut microbiota in triggering inflammatory vascular phenotypes. In times of spreading antimicrobial resistance, every unnecessary use of antibiotics should be carefully weighed up with the expected findings (67). As pointed out in this review, antibiotic treatment results in significant changes of host physiology, but the underlying mechanisms remain often unclear. Since no standardized antibiotic treatment regimen exists (68) and residual surviving microbiota differs dependent on the animal facility and housing conditions, the reproducibility of this experimental procedure is at stake (69, 70). Therefore, all the functional studies on the gut microbiota exploiting antibiotic treatment should at least mention the limitations of this technique and point out what kind of controls were included. In contrast, the GF mouse model remains the state-of-the-art approach for studying host-microbe and microbe-microbe interactions, since mono-colonization, minimal microbial consortia or humanized microbial consortia are standardized experimental approaches (71). Therefore, if GF mouse technology or gnotobiotic animal models are available and applicable, the use of antibiotic treatment should certainly be reconsidered.

## Author Contributions

All authors listed have made a substantial, direct and intellectual contribution to the work, and approved it for publication.

### Conflict of Interest Statement

The authors declare that the research was conducted in the absence of any commercial or financial relationships that could be construed as a potential conflict of interest.
